# Factors associated with blood pressure disorders in Afro-descendant children and adolescents

**DOI:** 10.1186/s12887-019-1626-0

**Published:** 2019-07-20

**Authors:** Fernando Rodrigues Peixoto Quaresma, Erika da Silva Maciel, Francisco Winter dos Santos Figueiredo, Fernando Adami

**Affiliations:** 1 Laboratory of Epidemiology and Data Analysis, Faculdade de Medicina do ABC, Av. Lauro Gomes, 2000, Santo André, São Paulo Brazil; 2 Federal University of Tocantins, Campus Palmas, Quadra 109 Norte, Avenida NS15, ALCNO-14 - Plano Diretor Norte, Palmas, TO Brazil; 3 Federal University of Tocantins, Miracema Campus, Av. Lourdes Solino, 195 - St. Sussuapara, Miracema do Tocantins, TO Brazil

**Keywords:** Blood pressure, Nutritional status, Physical activity, Anthropometry, Dyslipidemia, Social vulnerability

## Abstract

**Background:**

Hypertension (AH) is an emerging disease that has rapidly increased in the last decades throughout the world. The increase in blood pressure (BP) is observed with growth and development and, although the manifestation of the disease is rare in childhood and adolescence, its occurrence is increasing and the causes are likely to be from different combinations of factors. Afrodescendants have been consistently observed in many populations, including Brazil, which has the largest population of Afrodescendants outside Africa; nevertheless, data is scarce on the disease in children and adolescents. In this study, we investigated BP disorders in children and adolescents of “Quilombola” populations of the state of Tocantins, northern Brazil, and determined the disease occurrence with some factors, namely food consumption, body composition, anthropometric measures, and biochemical data.

**Methods:**

We carried out a cross-sectional study with 67 children aged 10–17 years, comparing the variables studied between the normotensive and non-normotensive groups, using the Chi-square test for qualitative variables and the appropriate tests, according to data adherence to the Gaussian distribution for the quantitative variables. High blood pressure was defined as mean systolic or diastolic blood pressure ≥ 90 percentile for age, height, gender.

**Results:**

The rate of adolescents with BP disorders was 19.4% (prehypertension 14.9% and hypertension 4.5%). There were no significant differences between the sexes for high blood pressure. In the Poisson regression analysis, the high fat percentage was associated with elevated blood pressure (*p* = 0.021) for adolescents. Similar associations were observed for non-HDL-c (*p* < 0.001) and low calcium intake (*p* = 0.015).

**Conclusion:**

Most children and adolescents in “Quilombola” communities had normal blood pressure. However, higher levels of dyslipidemia and low calcium intake are factors associated with prehypertension in the population studied with high BP.

## Background

The term “quilombo” is associated to a place where communities of black people dwell in Brazil. In their history, black people have faced cruelty, fled from urban areas, and struggled for human rights. In their fight for freedom, they were organized in groups, forming the communities of “quilombola” [1]. In 2017, “quilombolas” accounted for roughly 1 million people in Brazil [2], facing numerous difficulties, such as social prejudice and health discrepancies [3, 4].

Most “quilombolas” live in precarious conditions, without basic sanitation and water quality, with limited access to health services, hindering their humanized assistance with quality [5].

In Brazil, 21.4% of the population shows the systemic arterial hypertension (SAH) [6] disease, and this number is expected to continue to increase in the next years, according to the World Health Organization (WHO) [7]. There is an alert for a higher increase [8, 9] among the black population. Brazil has the largest population of Afro-descendants outside Africa [9, 10], this requires a priority in the research, prevention and treatment actions.

Other factors for differences in blood pressure disorders in ethnic groups are associated to demographic and socioeconomic components, such as education, income and financial stress that influence the prevalence of SAH in these populations [10, 11].

In children and adolescents, SAH has become frequent and worrisome, as the monitoring of health conditions in children and adolescents is different from that in adults, especially in terms of symptomatology [12].

The prevention of cardiovascular diseases in adulthood [13, 14] and risk factors in these groups [11, 15] requires the monitoring of blood pressure and people’s behaviors.

SAH is one of the main health problems worldwide; thus, early diagnosis is essential for SAH control and prevention of its secondary injuries. The “quilombola” communities have been affected by health problems of a vulnerable population. Thus, this research investigated the relationship between individual characteristics (gender, age, anthropometry, body composition, blood pressure, biochemical analysis) and lifestyle (dietary intake of sodium, calcium and physical activity level) in BP disorders in adolescents in communities of “quilombola”.

## Methods

### Study design

A cross-sectional study was carried out according to STROBE statement [16]. The variables of exposure were socio-demographic characteristics and lifestyle, and BP disorders in “quilombola” adolescents were the outcome variable.

### Setting

The study was carried out in a “quilombola” community in the state of Tocantins, northern Brazil, from June 2015 until November 2016.

The data were collected at facilities of the communities (schools, health units and “quilombola” association place), with the structure (rooms for examinations and interviews) adequately adapted for the research. The interviewers were previously trained and senior researchers with experience in studies on vulnerable communities accompanied the collections.

Data from the Information System on Remnant Communities of “Quilombo” register in Brazil, in 2017, about 2,394 “quilombola” communities. The state of Tocantins has 37 recognized “quilombola” communities and holds the 6th place regarding the number of “quilombola” communities [17].

### Participants

“Quilombola” adolescents, living in five “quilombolas” communities in the state of Tocantins, Brazil. The communities have 121 adolescents from 10 to 17 years old. Of this total, approximately 12 reside in the “*Córrego Fundo community”*; 18 in “*Manoel João”*; 28 in “*Malhadinha”*, (municipality of “*Brejinho de Nazaré”*); 39 in “*Barra da Aroeira”* (municipality of “*Santa Teresa”*) and 24 in “*Morro de São João”* (municipality of “*Santa Rosa”*).

The study included all adolescents (*n* = 121) between 10 and 17 years of age, who accepted to participate in the study after consent of parents or guardian (*n* = 73) and completed all collection steps (*n* = 67).

### Variables

For the analysis of this study, quantitative variables were considered (Table 1), as follows:

**Table 1:** Demographics, anthropometrics, body composition, blood pressure, physical activity levels, nutrient intake and biochemical analysis

Variables	Features
Gender	Male/Female
Age	Age in years
Anthropometrics	Height (cm)
	Weight (kg)
Body composition	BMI (kg/m^2^)
	Fat percentage (%)
Blood pressure	Normotension
	Pre-hypertension
	Hypertension
Physical activity (up to 13 years old)	METs (Meatabolic Equivalente Task)
Physical activity (> 13 years old)	METs
Nutrient intake	Sodium
	Total lipids
	Calcium
Biochemical analysis	Fasting glycaemia
	Non-HDL-c Cholesterol

### Data sources/ measurement

#### Anthropometrics

To measure the variables height (H) and weight (W), we used a wall stadiometer Seca 206® and a digital scale Hethmeter®, previously calibrated. The Body Mass Index (BMI) was classified for participants in terms of thinness, leanness, eutrophic, overweight, obese and severe obese, calculating the percentiles by age [18].

#### Body composition

We used Tetrapolar® [19] Electric Bioimpedance, which consists of a method that conducts a low intensity electricity through the body and measures indicators, such as metabolic rate and fat percentage, among others.

The fat percentage was classified for participants as very low up to 6% for boys and up to 12% for girls following, respectively, low from 6.01 to 10% and 12.01 to 15%, ideal from 10.01 to 20% and 15.01 to 25%, moderately high from 20.01 to 25% and 25.01 to 30%, high from 25.01 to 31% and 30.01 to 36%, and very high greater than 31.01% and greater than 36.01% [20].

#### Systemic blood pressure

We used the protocol of the VII Brazilian Guideline of Hypertension [21] to measure BP. We used a Tycos® mercury manometer with three different sizes of clamps (adult, adolescent, child) and a Littman® pediatric stethoscope. Systolic blood pressure (SBP) was determined by the appearance of Korotkoff sounds (K1). The fifth sound of Korotkoff (K5) was the definition of diastolic blood pressure (DBP).

BP disorders in children aged 10 to 12 years and adolescents aged 13 to 17 years were defined as SBP or Diastolic blood pressure (DBP) ≥ 90 percentile and hypertension was defined as SBP or DBP ≥ 95 percentile, according to the most recent guideline [22]. The formulas were based on the relationship between systolic BP (SBP) and diastolic BP (DBP) ages, heights, genders, according to a study that presents better evidence to identify high BP in children and adolescents [23].

#### Physical activity level

To assess the Physical Activity Level (PAL), two instruments were used: for the children at 10 to 13 years of age, we used the PAL Assessment Questionnaire and Sedentary Behavior [24], and for the participants above 14 years of age, the International Physical Activity Questionnaire (IPAQ) long version [25] was used.

The following criteria were used to classify PAL for participants 10 to 13 years of age: sedentary, if the total PAL per week was < 600 MET (Metabolic Equivalent); irregularly active, from 600 to < 1,500 MET/week; active, from 1,500 to 2,900 MET/week; and very active, if > 3,000 MET/week. Participants over 13 years of age were classified as sedentary if the total PAL per week was < 150 MET; irregularly active, from 150 to 630 MET/week; active, from 631 to 3,149 MET/week; and very active, if > 3,150 MET/week [26].

#### Food consumption

We used a record of 24 h that was applied by nutritionists previously trained through face-to-face interviews to quantify nutrient intake. For this study, only the usual consumption of sodium, calcium and lipids was calculated. The estimate was calculated in the Dietwin® software. The classification was performed using Dietary Reference Intakes [27] according to reference values for age and gender of the participant, which defines 1,5 g/day for daily (d) intake sodium and 1,3 g/day of calcium.

#### Biochemical blood analyses

Fasting glycaemia was evaluated by collecting 5 mL of blood in a tube without anticoagulant by venipuncture after fasting for 8 to 10 h. Non-HDL-c (High-density lipoprotein cholesterol) was calculated after analyzing cholesterol fractions quantified by the colorimetric enzymatic method with a fully automated spectrophotometer reading. The fractions of LDL-c (Low-density lipoprotein) and VLDL-c (Very low-density lipoprotein) were calculated (Friedwald formula) [28].

The non-HDL-c cholesterol fraction was calculated by the difference between total cholesterol and HDL-c. Total cholesterol is the total sum of potentially atherogenic plasma particles such as VLDL-c, IDL-c (Intermediate low-density lipoprotein) and LDL-c. The result of this calculation provides a better risk estimate compared to LDL-c, especially in cases of hypertriglyceridemia associated to diabetes, metabolic syndrome or renal disease [29].

### Bias

To reduce the risk of bias, there was previous training for instrument application, collection of exams, anthropometric and BP assessments, and the same researcher measured BP twice, according to recommendations [21].

To reduce information bias, an electronic form of data collection was created in Epi info 7.2® to build the databank. All data were validated in duplicate and in case of divergence between the data, a third researcher was consulted.

### Study size

Due to the scarce references about factors associated with hypertension in “quilombola” adolescents, non-probabilistic sampling was chosen for convenience, a sample composed of 121 adolescents living in the communities during the study.

### Quantitative variables

For the statistical calculations, we used gross data of variables fat percentage, nutrient intake, fasting glucose and non-HDL-cholesterol. For variables nutritional status, blood pressure, physical activity level, we used the classifications according to recommendations for height, gender and age.

### Statistical methods

After the standard descriptive statistical analysis, we compared the variables studied between the normotensive and non-normotensive groups (composed of individuals with prehypertension and hypertension) using the Chi-square test for qualitative variables and the appropriate tests, according to with data adherence to the Gaussian distribution. The Shapiro-Wilk test was used to analyze the normality of distribution.

The Poisson regression with robust variance was used to estimate the prevalence ratio grossly and in a multivariate way, following the *stepwise forward* strategy. The level of confidence was 5%. The program used was Stata® (StataCorp, USA) 11.0.

## Results

**Fig. 1:**
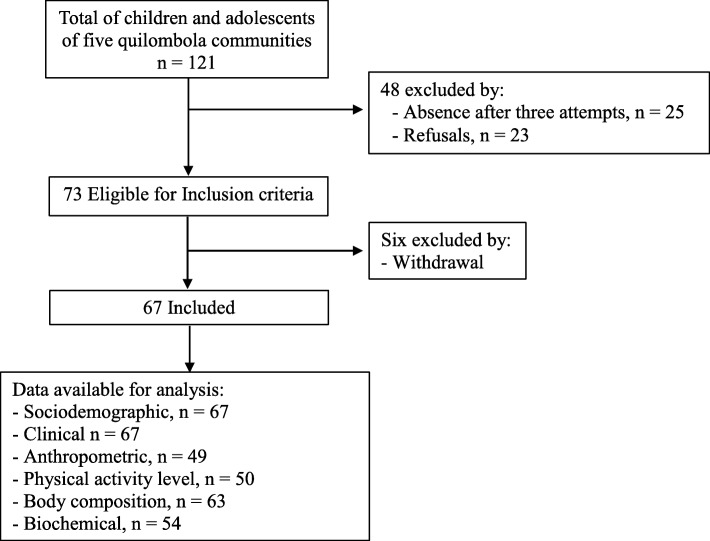
Flowchart of the study steps

Among 121 adolescents eligible for the inclusion criterion of the five “quilombola” communities, 67 subjects participated. Fifty-four subjects were considered absence losses after three collection attempts (21%), refusals (19%) and withdrawal (5%) in any of the stages. The greatest loss of data available for the analysis occurred with anthropometric data (Fig. 1).

Most subjects were male (*n* = 34, 50.7%), eutrophic (*n* = 37; 75.5%) with an average age of 13 years. Sedentary or irregularly active participants prevailed. Table 2 consolidates the main characteristics on demography, lifestyle, biochemical, and body composition. BP disorders were recorded in 19.4% and pre-hypertension in 14.9% (ranging from 6.1 to 23.7%), while hypertension was found in 4.5% (ranging from 0.6 to 9.6%) of adolescents (Table 2).

**Table 2:** Demographic characteristics of “quilombola” adolescents of Tocantins

Features	N	% (CI 95%)
Gender		
Female	33	49.3 (37.0; 61.5)
Male	34	50.7 (38.5; 63.0)
Nutritional status		
Thinness	6	12.2 (2.7; 21.8)
Eutrophy	37	75.5 (63.0; 88.0)
Overweight	4	8.2 (0.2; 16.1)
Obesity	2	4.1 (1.6; 9.8)
Physical activity level (up to 13 years old)		
Sedentary	39	100 (100.0; 100.0)
Irregularly active	–	–
Active	–	–
Very active	–	–
Physical activity level (> 13 years old)		
Sedentary	–	–
Irregularly active	4	36.4 (2.4; 70.3)
Active	7	63.6 (29.7; 97.5)
Very active	–	–
Blood pressure		
Normotension	54	80.6 (70.9; 90.3)
Prehypertensive	10	14.9 (6.1; 23.7)
Arterial hypertension	3	4.5 (0.6; 9.6)
Age	Mean	CI95%
	13.0	12.0; 14.0
Nutrient intake		
Sodium^a^	2,339.8	1,667.2; 2,857.7
Calcium^a^	255.5	179.5; 302.5
Biochemical Data	Mean	CI (95%)
Glycaemia^b^	87.5	81.0; 90.1
Non-HDL-c Cholesterol^b^	71.8	56.3; 87.2

^a^Recommended Dietary Adequate Intakes: sodium 1.00 mg/day, calcium 1,300 mg/day [27]

^b^ Brazilian Guidelines of Dyslipidemia and Atherosclerosis Prevention. Values for teenagers from 10 to 19 years of age: Non HDL-c: < 120 [29]

In the multivariate analyses, the characteristics that influenced BP disorders were the nutrient intake and biochemical data (Table 3). The non-HDL-c cholesterol (PR = 1.05, ranging from 1.03 to 1.08, *p* < 0.001), calcium intake (PR = 1.005, ranging from 1.001 to 1.01, *p* = 0.015) and fat percentage (PR = 1.25, ranging from 1.03 to 1.51, *p* = 0.021) were factors that influenced the increase in blood pressure, when they increased (Table 4).

**Table 3:** Association between body features of “quilombola” adolescents, according to systemic arterial hypertension, Tocantins

Features	Blood Pressure Classification		P*	Poisson Regression	
	Normotension (*N* = 54; 80.6%)	High blood pressure / Hypertension (*N* = 13; 19.4%)		RP (CI 95%)	p**
Gender	n (%)				
Female	27 (81.8)	6 (18.2)	0.803	Ref.	Ref.
Male	27 (79.4)	7 (20.6)		1.13 (0.38; 3.37)	0.823
Nutritional status	130 (12.0; 14.0)	12.0 (10.0; 14.6)	0.382	−1.0 (−3.7; 1.8)	0.471
Eutrophy	32 (86.5)	5 (13.5)	0.476	Ref.	Ref.
Overweight / Obesity	4 (66.7)	2 (33.3)		2.46 (0.48; 12.7)	0.281
Thinness	5 (83.3)	1 (16.7)		1.23 (0.14; 10.6)	0.848
	Mean (CI 95%)		P***	Mean Difference (CI 95%)	P****
Age	13.0 (12.0; 14.0)	12.0 (10.0; 14.6)	0.382	−1.0 (−3.8; 1.8)	0.471
Blood Pressure (mmHg)					
Systolic	90.0 (90.0; 100.0)	110.0 (97.1; 120.0)	0.009	20.0 (−5.2; 45.2)	0.118
Diastolic	60.0 (60.0; 60.0)	80.0 (70.0; 80.0)	< 0.001	20.0 (−2.2; 42.2)	0.077
Body composition					
Height (in)	155.4 (151.9; 158.0)	149.2 (135.6; 160.9)	0.173	−6.2 (−14.0; 2.5)	0.160
Weight (kg)	45.0 (40.6; 47.8)	39.1 (29.6; 55.2)	0.262	−6.4 (−16.4; 3.6)	0.206
Fat percentage (%)	17.6 (15.6; 21.5)	18.0 (10.4; 22.8)	0.892	0.1 (−7.4; 7.6)	0.979
Nutrient intake					
Sodium (g)	2444.1 (1337.6; 3090.8)	1987.5 (862.9; 3821.5)	0.819	− 456.5 (− 2178.6; 1265.6)	0.597
Calcium (mg)	220.2 (150.9; 296.8)	309.9 (192.0; 433.9)	0.181	89.7 (−75.3; 254.7)	0.281
Biochemical Data					
Glycaemia	88.0 (81.0; 93.0)	85.0 (62.4; 94.8)	0.333	−3.0 (−16.8; 10.8)	0.663
	Mean (CI 95%)		p*+	Mean Difference	p*+
Non-HDL-c Cholesterol	60.7 (47.5; 75.8)	102.0 (55.9; 148.1)	0.013	−40.3 (−71.3; −9.4)	0.013

*Ref* Category of reference, *CI 95%* Confidence interval at 95%; *Chi-square; ** Mann-Whitney Test; *** Mann-Whitney; ****Interquartile Regression; p* + = *Student* t test

**Table 4:** Factors associated to blood pressure disorders in male “quilombola” adolescents through the Poisson regression, Tocantins

Features	Poisson Regression	
	β (CI 95%)	*p*
Non-HDL-c Cholesterol	1.05 (1.03; 1.08)	< 0.001
Calcium	1.005 (1.0001; 1.01)	0.015
Weight	1.07 (0.97; 1.18)	0.195
Male Gender vs Female Gender^a^	3.39 (0.20; 56.8)	0.395
Age	0.83 (0.57; 1.23)	0.363
Fat percentage	1.25 (1.03;1.51)	0.021

*CI 95%* Confidence interval at 95%, *PR* Prevalence ratio; ^a^Female Gender is the reference category

## Discussion

Our findings showed non-HDL-c, cholesterol excess and low consumption of dietary calcium, as the main causes of BP disorders in “quilombola” adolescents.

For the WHO, cardiovascular diseases are the main death cause worldwide and more people die annually from these diseases than from any other cause, and three-quarters of those deaths occur in low- and middle-income countries. Dyslipidemia represents one of the most significant risk factors of cardiovascular disease [30].

The average serum concentration of non-HDL-c among participants in the study sample was 102.0 mg/dL (55.9 to 148.1) higher in participants with BP disorders.

The results show that adolescents without changes of BP have less concentration of non-HDL-c cholesterol (with an average difference of − 40.3 (− 71.3 to − 9.4)) compared to those with changes in BP. Although we did not find studies that assessed risk factors for BP disorders adolescents taking into account non-HDL-c, the results corroborate with other studies that assessed associations to heart disease using the same variables of this study. Some researchers have pointed out that non-HDL-c is one of the best predictors of atherosclerotic risk in children and adolescents [31, 32] because it is more strongly associated to lesions in the abdominal and coronary aorta than to other lipid fractions [33], in addition to association to metabolic diseases [34, 35].

The results for body composition confirm recent findings of Hudson et al. [36] for obese adolescents of 12–19 years of age in the United Kingdom, suggesting that BP disorders were low, despite the positive association of fat percentage with arterial stiffness and adiposity.

Previous evidence [37–40] suggests that adiposity may influence vasculature, leading to hypertension. Other studies also reported that overweight and obesity increased the risk of hypertension 1.22 (95% CI: 1.05, 1.42) and 1.78 (95% CI: 1.33, 2.37), respectively [41].

The divergence between the results suggests the need for monitoring these variables during life. In this research, BP disorders in adolescents are associated to obesity and indicates that the social gradient grows toward socially disadvantaged segments, a scenario observed in populations with low economic and schooling levels, corroborating with environmental inequities [42, 43] and health services [44], *sine qua nom* gears for health promotion and prevention of cardiovascular diseases (CVD).

Studies report that low calcium intakes were inversely related to BP disorders and that increased calcium intake minimizes these risks [45]. Our findings show a significant association between low dietary calcium intake and BP disorders in “quilombola” adolescents with dietary calcium intake lower than 1300 mg/day.

Conversely, Kong et al. [46] assessed the risks of all-cause mortality, CVD, Cerebral Vaccine Accident (CVA), or bone fracture due to inadequate dietary calcium, using an epidemiological outline in Korea, with 2153 women and 2158 men. The authors concluded that women with higher calcium intakes were associated to a reduced risk of CVD, but not to stroke or bone fracture, and in men, there was no significant correlation.

There were no significant effects for sodium intake with significant change in BP levels. Both SBP and DBP were slightly higher among BP-free participants who consumed more than 2,300 mg/day of sodium for adolescents who presented changes and consumed less than 2,300 mg/day (90 mmHg vs. 110 mmHg for SBP and 60 mmHg vs. 80 mmHg for DBP, respectively). Similarly, Padilha et al. [47] reported that participants with hypertension showed lower sodium consumption in the diet than those with normal BP. A meta-analysis showed that reductions in dietary sodium lead to modest reductions in children blood pressure [48]. Lower of sodium intake during childhood also appears to protect against increase of blood pressure [49]. Our results may be due to the great variability of using a single 24-h record of dietary data, as well as the lack of temporality. The actual rate of sodium consumption may have been underestimated for the population of this study. In addition, in order to keep healthy BP levels throughout life, there is strong support for initiatives that seek to reduce sodium intake among children [50].

The findings of this study are subject to some limitations. First, several attempts to collect data from all adolescents in the communities were performed, however, there were data losses that reduced the sample size. Furthermore, the lack of temporality among the variables in a cross-sectional study was an important limitation to the study. This reduced sample size may have been a limitation, given the extrapolation ability of the results found in this study. However, no previous study attempted to assess BP disorders in adolescents of these “quilombola” communities, which makes this study an important parameter to estimate the sample size adequate for other studies.

## Conclusion

The results of this study suggest that the early deregulation of nutrient intake and excess of non-HDL-c cholesterol can represent important risk factors of prehypertension in the “quilombola” population. The results indicate the importance of a favorable combination between physical activity and nutrition environments in places where children and adolescents live to sustain a healthy course and behaviors regarding BP disorders.

## Data Availability

The datasets used and/or analyzed during the current study are available from the corresponding author on reasonable request.

## Abbreviations

BMI: Body Mass Index
BP: Blood pressure
CI: Confidence interval
CVA: Cerebral Vaccine Accident
CVD: Prevention of cardiovascular diseases
DBP: Diastolic blood pressure
IPAQ: International Physical Activity Questionnaire
LDL-c: Low-density lipoprotein
MET: Metabolic Equivalent
N: Number
Non-HDL-c: High-density lipoprotein cholesterol
PAL: Physical activity level
RP: Prevalence ratio
SAH: Systemic arterial hypertension
SBP: Systolic blood pressure
STROBE: Strengthening the Reporting of Observational Studies in Epidemiology
VLDL-c: Very low-density lipoprotein
WHO: World Health Organization
